# Intimate partner violence and excess fertility among women of reproductive age in Malawi

**DOI:** 10.1371/journal.pone.0297959

**Published:** 2024-01-26

**Authors:** Sufia Dadabhai, Laura Quaynor, Antonio Bandala-Jacques, Linly Seyama, Md Hafizur Rahman, Richard Phiri, Michele R. Decker, Taha E. Taha

**Affiliations:** 1 Department of Epidemiology, Johns Hopkins Bloomberg School of Public Health, Baltimore, Maryland, United States of America; 2 Department of Advanced Studies in Education, Johns Hopkins School of Education, Baltimore, Maryland, United States of America; 3 Kamuzu University of Health Sciences-Johns Hopkins Research Project, Blantyre, Malawi; 4 Department of International Health, Johns Hopkins School of Public Health, Baltimore, MD, United States of America; 5 Malawi National Statistics Office, Zomba, Malawi; 6 Department of Population, Family & Reproductive Health, Johns Hopkins Bloomberg School of Public Health, Baltimore, Maryland, United States of America; Hawassa University College of Medicine and Health Sciences, ETHIOPIA

## Abstract

**Purpose:**

Gender inequity and adverse health outcomes continue to be of concern among women in sub-Saharan Africa. We determined prevalence of intimate partner violence and excess fertility (having more children than desired) in reproductive age women in Malawi. We also explored factors associated with these outcomes and with spousal fertility intentions.

**Patients and methods:**

In a cross-sectional study, a total of 360 women and 410 men were recruited using multi-stage sampling from communities in a peri-urban setting in Blantyre District, Southern Malawi in 2021. Women and men were separately interviewed by trained study workers using a structured questionnaire. In addition to descriptive analyses, we used univariate and multivariate logistic regression models to assess associations of risk factors with the outcomes of intimate partner violence and excess fertility.

**Results:**

Among women, lifetime prevalence of intimate partner violence was 23.1%, and excess fertility was experienced by 25.6%. Intimate partner violence was associated with male partners alcohol consumption (adjusted odds ratio 2.13; P = 0.019). Women were more likely to report excess fertility if they were older (adjusted odds ratio 2.0, P<0.001, for a 5-year increase). Alcohol consumption by the male partner (adjusted odds ratio 2.14; P = 0.025) and women being able to refuse sex with their male partner (adjusted odds ratio 0.50; P = 0.036) were associated with discordant fertility preferences.

**Conclusions:**

Intimate partner violence, excess fertility, and social and health inequities continue to be prevalent in Malawi. These data suggest the underlying proximal and distal factors associated with these adverse outcomes such as alcohol consumption may be addressed through education, couple interactive communication, and community dialogue. To ensure sustainability and effectiveness, strong leadership involvement, both governmental and non-governmental, is needed.

## Introduction

The health and well-being of women in Malawi is influenced by a host of gender equity factors across the socio-ecologic model [1]. Women’s degree of disempowerment, in terms of control over sexual activity and reproductive preferences can impact their health, their children’s health, and that of other family members. Fertility remains high in Malawi, and is decreasing at a slow pace [2]. Both proximal and distal factors influence fertility [3] and fertility intentions among women, including reproductive history, use of family planning, family support, and history of intimate partner violence (IPV) [2,3–6]. Malawi’s HIV epidemic also shapes women’s fertility intentions and outcomes: there have been concerns that HIV diagnosis may prompt women to try to hasten pregnancies in fears that they may not be able to do so in the future [7,8], antiretroviral treatment may change fertility [9], and a host of biological and indirect behavioral factors may also affect fecundity in women living with HIV in Sub-Saharan Africa [10].

Domestic violence is prevalent in the Malawi setting. Per the 2015 Malawi Demographic and Health Survey (MDHS) an estimated 34% of women reported experiencing physical domestic violence since age 15 in Southern Malawi, and 17% reported ever having sexual violence [11]. Among married women, alcohol consumption by the spouse, ethnicity and women’s working status have been found to be associated with IPV [12]. Broader contributors to IPV include demographic and socioeconomic background characteristics including age, parity, education, and household economic status [13].

Distal gender equity factors that contribute to IPV include economic security, relationship quality, motivation for parenthood, and decision-making autonomy. Economic security refers to women’s dependence on their spouse for resource support. Although economic security has improved in Malawi in recent years, it has been tenuous for decades, and wage earnings are limited, especially for female workers [14]. As women who have living children are more likely to work, it is possible that economic security might force women into the workforce, particularly into the agrarian sector [14]. For partner relationship quality, partner’s positive assessments of their relationship, as determined by communication and intimacy, enhance their concordance of family size and pregnancy planning, and reduce risk of domestic violence [15]. Regarding motivation for parenthood, assessing gender imbalances in individual motivations for parenthood might help understand persisting high levels of fertility. Lastly, autonomy in decision making (such as autonomy to obtain own healthcare, travel, go out, or visit relatives) is a critical factor for women’s health as they experience the achievement of their choices [16].

Against this backdrop of potentially shared gendered equity threats to both IPV and excess fertility, the objectives of our study were to describe lifetime IPV prevalence, excess fertility, and spousal discordance in fertility preferences; and their individual and relational determinants, among women and men living in a peri-urban setting of Blantyre, Malawi. This study provides more current information on IPV and its related factors in a localized community compared to the MDHS which was conducted in 2015 [11].

## Material and methods

### Study design

We conducted a cross-sectional, multi-stage sampling study where participants were recruited from communities in the Lunzu area around Blantyre in the Southern Region of Malawi. Blantyre (population of more than 800,000) is the second largest city in Malawi and is mostly known as a commercial and financial center. Women and men (including couples) were enrolled in the survey after appropriate counseling by trained study workers and signing a written informed consent form. Participating women and men completed a structured questionnaire (face-to-face interview) administered by study workers with adequate knowledge of the community. The data collection was conducted at each participant’s home, separately for the woman and the man. This study was conducted during the period 2020–2021. The study was approved by the Johns Hopkins Bloomberg School of Public Health Institutional Review Board (IRB) (Study Title: “Understanding and Promoting Health Equity Among Women of Reproductive Age in Peri-Urban Blantyre, Malawi” IRB No.: 12795) and by the Malawi College of Medicine Research and Ethics Committee (# P.08/20/3111).

To produce timely, reliable, and representative information, a multi-stage study design approach was employed to identify potential participants. The Blantyre district in Malawi comprises Blantyre city and Blantyre rural areas; these are demarcated into standard enumeration areas (SEAs). Using ArcGIS software, a 4.5 km buffer was specified and implemented in the Blantyre peri-urban area of Lunzu trading center. The extraction yielded 28 SEAs (primary sampling units) in the Traditional Authority Kapeni area. The population sizes of the SEAs vary; the majority range from 150 to 250 households. A sample of 10 SEAs were randomly selected from the 28 SEAs yielding 400 households. Subsequently, 40 households per SEA were selected using systematic sampling technique. Prior to the data collection phase, an exhaustive list of households was extracted from the dwelling unit database of 2018 Malawi Population and Housing Census. Household heads, sex and geo-position were extracted from the population database and merged with dwelling units. Married participants 18 years (lower age for consenting) and older, living in the same household, and who were willing to sign a consent form and be interviewed separately were eligible. For number of necessary participants, we estimated a sample size of 350 women and 400 men across all households (including 10% nonresponse; statistical power >80%). More married men than women were included based on information from the previous MDHS conducted in 2015 [11] which showed an average of 0.95 eligible women aged 15–49 and 0.90 eligible men aged 15–54 per household, and response rates of 0.99 for females and 0.95 for males. The MDHS also showed 55.5% of women reported having no more children and 20.1% of ever-married women reported experiencing physical or sexual violence; for men 50% reported wanting no more children.

Initially, the study was introduced to the communities in the selected areas via general talks with village chiefs. Households in the selected areas were approached and provided general information about the study to gauge their interest and willingness to know more about the study. The questionnaire we used was comparable in many sections to the latest version of the 2015 Malawi Demographic and Healthcare Survey (MDHS) [11]. The questionnaires consisted of multiple sections for women and men, and primarily focused on reproduction, contraception, marriage and sexual activity, fertility preferences, IPV, and health issues. Study workers were trained to observe confidentially and ensure the environment within the premises was appropriate to avoid presence of other people and distractions. Data were directly entered in hand-held laptops using OpenDataKit (ODK) and downloaded on daily basis at a central location at the Johns Hopkins Research Project offices in Blantyre, Malawi. The study conduct was coordinated and supervised by a designated resident investigator (LS).

### Analysis plan

The outcome variables included IPV (physical or sexual; ever), excess fertility, and spousal discordance in fertility preferences. For physical IPV, we used a subset of questions focusing on specific actions (e.g., pushed/shaken/something thrown; slapped; twisted arm/pulled hair; punched; kicked/dragged/beaten; choked/burned; threatened with a weapon), based on standard DHS assessments. For sexual IPV we assessed whether the woman reported any of the following: not wanting to have sex at that time, feeling pressured by her husband/partner to have sex then, being forced to have sex, or feeling at risk of physical violence if she declined to have sex. These items were adapted from the 2015 MDHS [11]. Excess fertility was defined as having more children than desired [17]. For this purpose, we added the total number of living children each woman had and compared them to what they responded in the question about how many children they would ideally like to have if they could go back in time. We defined spousal discordance in fertility preferences as a disagreement in the answer to the question of wanting more children within each couple.

For the exposure variables, we estimated age as the time difference in years between birth and time of survey. We dichotomized reported highest level of education for the woman into secondary or higher versus primary or less. We obtained information on having electricity at home as a better measure of socioeconomic status in this setting. We defined recent HIV status as having taken an HIV test in the last 12 months prior to the survey. We defined effective family planning as using one of the following family planning methods: injectables, pills, implants, an intrauterine device (IUD), or hysterectomy. We used standard DHS assessments for decision-making autonomy (making decisions alone or jointly with spouse about healthcare, household purchases, and visits to relatives), and economic security (deciding how to spend money either alone or as a joint decision with spouse). Women were asked standard items on justification of violence, specifically if a male partner is justified in hitting or beating her in some specific situations (going out without telling him, neglecting the children, arguing with him, refusing to have sex with him or burning the food). Women who answered yes to any of these questions were considered having justification of violence.

We first performed descriptive analyses of the household survey and of the gender-specific surveys. For numeric variables we present median and interquartile range (IQR). For categorical variables we present counts and proportions. We stratified the results by gender in most of the analyses. We performed logistic regression analyses to identify factors associated with IPV and excess fertility after controlling for other factors. For the spousal discordance analysis, we performed three separate logistic regression models. First, to account for coupling, we clustered spouses by a shared ID and used robust standard errors. We then performed two separate analyses among women’s and men’s responses. In all multivariate analyses the choice of risk factors to include in the models was based on the conceptual framework we assumed, biological and epidemiological factors, and statistical significance. We evaluated correlation of the independent variables using the variance inflation factor. For the regression models, we presented odds ratios along with 95% confidence intervals.

All analyses were performed on R Version 4.1.3 (R Core Team, Vienna, Austria) via Rstudio version 2022.2.1.461 (Rstudio Team, Boston, MA). All data were anonymized at the analysis stage and no data with identifiers were available upon closure of recruitment and final data management.

### Inclusivity in global research

Additional Information regarding the ethical, cultural, and scientific considerations specific to inclusivity in global research is included in the Supporting Information.

## Results

A total sample of 360 women and 410 men were included in this survey. Table 1 shows the characteristics of these participants. Median age for women was 31 years (IQR 23–39) and median age for men was 36 years (IQR 29–44). Participant’s most common highest level of completed education was primary school or less (63% for women and 55% for men, P = 0.067). In women, 95% reported having children, and in men, 91% reported ever having fathered a child (P = 0.040). From the household survey, 15.6% of women and 13.9% of men reported having electricity at home (P = 0.517). When asked about HIV testing in the last 12 months, 1% of women and 8% of men refused to answer (P<0.001). From the remainder, similar proportions of men and women reported being tested for HIV in the last 12 months prior to the survey (69.7% women and 63.9% men). Men were also more likely to report being a smoker than women (13% vs. 1%, P<0.001), and more likely to report being employed, excluding housework (85% vs. 53%, P<0.001).

**Table 1:** Sociodemographic characteristics of study participants, Lunzu area, Blantyre, Malawi, 2020–2021.

Variable	Women (N = 360)	Men (N = 410)		P-value ^c^
**Age groups–N(%)**				
<25	101 (28.1)	57 (13.9)		**<0.001**
25–29	72 (20.0)	53 (12.9)		
30–34	49 (13.6)	65 (15.9)		
35–39	54 (15.0)	76 (18.5)		
40–44	45 (12.5)	64 (15.6)		
45–49	18 (5.0)	41 (10.0)		
≥50	21 (5.8)	54 (13.2)		
**Highest education level–N (%)**				
Primary or less	226 (62.8)	227 (55.4)		0.067
Secondary or higher	115 (31.9)	164 (40.0)		
Did not answer	19 (5.3)	19 (4.6)		
**Has ever had children–N (%)**				
Yes	342 (95.0)	374 (91.2)		**0.040**
**Uses effective family planning methods** ^**b**^ ^–^**N (%)**				
Yes	245 (69.4)	^a^	^a^	–
**Has electricity at home–N (%)**				
Yes	56 (15.6)	57 (13.9)		0.517
**Tested for HIV in last 12 months–N (%)**				
Yes	251 (69.7)	262 (63.9)		**<0.001**
No	106 (29.4)	117 (28.5)		
Did not answer	3 (0.8)	31 (7.6)		
**Smokes cigarettes–N (%)**				
Yes	2 (0.60)	55 (13.4)		**<0.001**
**Has health insurance–N (%)**				
Yes	4 (1.11)	8 (2.0)		0.347
**Has non-housework employment–N (%)**				
Yes	187 (53.0)	354 (86.3)		**<0.001**

Notes

a: Not asked in men.

b: Injectables, pills, implants, intrauterine devices (IUDs), or hysterectomy.

c: Chi-squared test.

Table 2 summarizes survey questions regarding IPV and fertility. From the 360 women, 23% (95% CI 18.7–27.4) reported ever experiencing any type of IPV (specifically, 12% physical violence, and 18% sexual violence). Overall, 7.5% women did not answer at least one of these questions. Thirty-three percent (33%) of women reported that their own mothers experienced physical violence themselves, and 30% reported that their spouses consume alcohol.

**Table 2:** Intimate partner violence and excess fertility among 360 women from Lunzu area, Blantyre, Malawi, 2021–21.

Variable	N	%
**Ever experienced physical IPV**		
Yes	43	11.9
No	292	81.1
Did not answer	25	6.9
**Ever experienced sexual IPV**		
Yes	64	17.8
No	274	76.1
Did not answer	22	6.1
**Ever experienced IPV (any)**		
Yes	83	23.1
No	250	69.4
Did not answer	27	7.5
**Participant’s mother experienced domestic partner violence**		
Yes	117	32.5
No	193	53.6
Does not know	40	11.1
Did not answer	10	2.8
**Thinks domestic partner violence is justified in some situations**		
Yes	56	15.6
No	303	84.2
**Male partner drinks alcohol**		
Yes	108	30.0
No	243	67.5
Did not answer	9	2.5
**Feels economically secure** ^**a**^		
Yes	169	46.9
No	191	53.1
**Has autonomy for decision-making** ^**b**^		
Yes	136	37.8
No	224	62.2
**Discussed contraception with a healthcare professional within 12 months**		
Yes	252	72.0
No	98	28.0
**Has more children than desired (excess fertility)**		
Yes	92	25.6
No	221	61.4
Did not answer	47	13.1
**Thinks women can participate in decision to use contraception**		
Yes	303	84.2
No	55	15.3
Did not answer	2	0.6

Notes

a: Makes decisions alone or jointly with spouse about healthcare, household purchases, and visits to relatives.

b: Decides how to spend money either alone or as a joint decision with spouse.

Overall, 16% of women and 5% of men (P<0.001, not shown) reported that partner violence is justifiable under certain conditions. In this setting, 47% of women responded positively to questions suggesting a feeling of economic security and 38% to questions suggesting a feeling of autonomy in decision making. When asked about contraception, 70% of women had discussed these with a healthcare professional within the last 12 months, compared to 14% of men (P<0.001, not shown), and 84% of women reported that men should be able to participate in contraception decisions. Finally, 26% of women reported having more children (excess fertility) than what they would ideally have if they could go back in time.

Table 3 shows results of the logistic regression analysis for the association of selected risk factors with IPV among women. In this model, in the univariate analysis, reported male partners consuming alcohol, and women’s father hitting their mothers were associated with statistically significant increase in IPV (more than two-fold); these univariate associations were also seen after controlling for other factors (both in direction and magnitude). Woman’s participation in contraceptive decision-making was associated with statistically significant lower risk of IPV in the univariate analysis (P = 0.03) but was not maintained after adjusting for other factors in the multivariate analysis. The other sociodemographic factors included in the model were not statistically significant.

**Table 3:** Logistic regression: Risk factors associations with lifetime IPV in women, Blantyre, Malawi.

	Unadjusted		Adjusted	
Variable	OR ^a^ (95% CI)	P	OR ^a^ (95% CI)	P
Age (5-year increments), continuous	0.93 (0.81–1.05)	0.252	0.86 (0.72–1.02)	0.083
Highest level of education ^b^	0.64 (0.36–1.10)	0.116	0.67 (0.35–1.25)	0.217
Has electricity, yes vs. no (ref)	1.56 (0.79–2.96)	0.443	1.72 (0.80–3.61)	0.157
Recent HIV test, yes vs. no (ref)	0.86 (0.50–1.49	0.586	0.75 (0.39–1.46)	0.393
Uses effective family planning, yes vs. no (ref)	0.99 (0.55–1.83)	0.970	0.91 (0.43–1.96)	0.805
Partner drinks alcohol, yes vs. no (ref)	2.93 (1.75–4.93)	**<0.001**	2.13 (1.13–4.01)	**0.019**
Thinks partner violence is justified, yes vs. no (ref)	1.61 (0.83–3.02)	0.148	0.97 (0.41–2.20)	0.945
Woman’s father used to hit woman’s mother, yes vs. no (ref)	2.43 (1.45–4.08)	**<0.001**	2.21 (1.21–4.02)	**0.009**
Woman participates in contraception decision, yes vs. no (ref)	0.50 (0.27–0.95)	**0.031**	0.57 (0.26–1.30)	0.178
Economic security, yes vs. no (ref)	1.61 (0.98–2.66)	0.062	1.31 (0.72–2.40)	0.374
Decision-making autonomy, yes vs. no (ref)	1.93 (1.16–3.20)	**0.011**	1.45 (0.78–2.70)	0.234

Notes

a: OR–odds ratio.

b: Secondary or higher vs. primary or less (ref).

Table 4 shows results of the logistic regression analysis for the association of risk factors with excess fertility among women. In the univariate analysis, increase in age was associated with statistically significant higher odds of reported excess fertility and higher level of education was associated with statistically significant lower odds of reported excess fertility. In the multivariate adjusted model, the statistically significant association with older age persisted but not with education. However, use of an effective family planning method showed a strong (OR = 2.76) statistically significant (P = 0.029) association with reported excess fertility in this cross-sectional study. We investigated this association further by examining the socio-demograhic charactertitics of women who used or not used an effective family planning method and did not observe women who used effective family planning methods to be older, more educated, have more children, want less children, or of higher socio-economic status. Other factors were not associated with excess fertility, including ever having IPV (P = 0.92).

**Table 4:** Logistic regression: Risk factor associations with excess fertility in women, Blantyre, Malawi.

	Unadjusted		Adjusted	
Variable	OR ^a^ (95% CI)	P	OR ^a^ (95% CI)	P
Age (5-year increments), continuous	1.89 (1.61–2.25)	**<0.001**	2.03 (1.64–2.58)	**<0.001**
Highest level of education ^b^	0.55 (0.30–0.95)	**0.036**	0.64 (0.31–1.27)	0.208
Has electricity, yes vs. no (ref)	1.32 (0.68–2.49)	0.403	1.02 (0.42–2.36)	0.967
Recent HIV test, yes vs. no (ref)	0.73 (043–1.23)	0.233	1.51 (0.74–3.17)	0.264
Uses effective family planning, yes vs. no (ref)	0.69 (0.39–1.26)	0.218	2.76 (1.14–7.11)	**0.029**
Partner drinks alcohol, yes vs. no (ref)	1.1 (0.65–1.85)	0.715	1.02 (0.50–2.08)	0.946
Thinks partner violence is justified, yes vs. no (ref)	0.49 (0.20–1.04)	0.081	0.87 (0.28–2.39)	0.790
Woman’s father used to hit woman’s mother, yes vs. no (ref)	1.09 (0.64–1.82)	0.750	0.89 (0.45–1.76)	0.749
Woman participates in contraception decision, yes vs. no (ref)	0.89 (0.44–1.92)	0.762	1.06 (0.39–3.04)	0.914
Ever domestic partner violence, yes vs. no (ref)	0.87 (0.48–1.56)	0.700	0.92 (0.42–1.98)	0.832
Economic security, yes vs. no (ref)	1.30 (0.80–2.12)	0.289	0.99 (0.50–1.91)	0.969
Decision-making autonomy, yes vs. no (ref)	0.79 (0.47–1.31)	0.366	1.38 (0.68–2.79)	0.371

Notes

a: OR–odds ratio.

b: Secondary or higher vs. primary or less (ref).

Table 5 shows results of the logistic regression models for the association of selected risk factors with spousal discordance in reported fertility preferences. Out of 323 matched couples, responses of 72 (22.2%) were discordant on fertility preference, and 251 were concordant. In the model that considered both participants’ responses (couples), none of the variables we included in this model were associated with statistically significant discordance in fertility preferences. In the models stratified by gender (Table 5), women using an effective family planning method (OR 0.41, P = 0.026), and those able to refuse sex with their partner (OR 0.50, P = 0.036) had a statistically significant lower likelihood of fertility discordance. Additionally in this model having a male partner who consumes alcohol (adjusted OR 2.14, P = 0.025), increased the likelihood of fertility discordance after controlling for other variables. In the model that examined men’s responses, increase in age was associated with lower odds of fertility discordance (OR 0.88, P = 0.038) and likewise men who reported having only one wife/partner showed a statistically significant lower risk of discordance in fertility preferences (0.21, P = 0.028).

**Table 5:** Logistic regression analyses showing the association of selected factors with spousal discordance in fertility preferences among couples in Blantyre, Malawi.

Variable	Unadjusted OR (95% CI) ^a^	P	Adjusted OR (95% CI) ^a^	P
**Model 1: Among couples**				
Age, 5-year increments	0.88 (0.79–0.97)	**0.014**	0.91 (0.79–1.04)	0.163
Has electricity, yes vs. no (ref)	0.86 (0.40–1.81)	0.697	0.97 (0.45–2.07)	0.930
Number of living children, 1–2 vs. 0 (ref)	1.58 (0.74–3.42)	0.240	1.46 (0.67–3.20)	0.345
Number of living children, 3–4 vs. 0 (ref)	1.77 (0.78–4.02)	0.175	1.85 (0.79–4.30)	0.155
Number of living children, 5+ vs. 0 (ref)	0.62 (0.25–1.53)	0.298	0.74 (0.28–2.00)	0.558
Has heard of family planning, yes vs. no (ref)	1.02 (0.57–1.85)	0.941	1.04 (0.55–1.94)	0.910
Maximum education level, primary versus none (ref)	0.58 (0.25–1.35)	0.210	0.46 (0.19–1.13)	0.090
Maximum education level, secondary+ versus none (ref)	0.81 (0.33–1.99)	0.639	0.54 (0.20–1.44)	0.218
Thinks IPV is justified, yes vs. no (ref)	1.07 (0.60–1.89)	0.822	0.85 (0.45–1.59)	0.610
**Model 2: Among women**				
Age, 5-year increments	0.86 (0.74–0.99)	**0.041**	0.80 (0.66–0.98)	**0.044**
Uses effective family planning, yes vs. no (ref)	0.87 (0.49–1.57)	0.628	0.41 (0.19–0.91)	**0.026**
Thinks contraception is a joint decision, yes vs. no (ref)	1.21 (0.68–2.20)	0.523	1.18 (0.65–2.23)	0.112
Concordant with partner on number of children wanted, yes vs. no (ref)	1.52 (0.91–2.60)	0.111	1.03 (0.52–2.04)	0.286
Has more children than desired, yes vs. no (ref)	0.58 (0.28–2.11)	0.112	0.70 (0.35–1.78)	0.596
Has experienced IPV, yes vs. no (ref)	1.40 (0.76–2.51)	0.272	0.91 (0.39–1.74)	0.641
Partner drinks alcohol, yes vs. no (ref)	1.85 (1.07–3.19)	**0.027**	2.14 (1.15–4.29)	**0.025**
Can refuse to have sex, yes vs. no (ref)	0.69 (0.45–1.20)	0.185	0.50 (0.26–0.96)	**0.036**
Thinks IPV is justified, yes vs. no (ref)	1.46 (0.73–2.79)	0.268	0.72 (0.29–1.64)	0.500
Economic security, yes vs. no (ref)	1.42 (0.84–2.41)	0.191	1.63 (0.87–3.11)	0.130
Decision-making autonomy, yes vs. no (ref)	1.41 (0.82–2.39)	0.208	1.01 (0.51–1.96)	0.971
**Model 3: Among men**				
Age, 5-year increments	0.88 (0.77–0.99)	**0.042**	0.88 (0.75–0.98)	**0.038**
Has discussed family planning with healthcare professional, yes vs. no (ref)	0.69 (0.30–1.44)	0.348	0.68 (0.28–1.47)	0.346
Thinks contraception is only a woman’s concern, yes vs. no(ref)	0.78 (0.38–1.52)	0.491	0.78 (0.35–1.65)	0.534
Has only one wife, yes vs. no (ref)	0.22 (0.05–0.84)	**0.025**	0.21 (0.05–0.86)	**0.028**
Believes condoms reduce HIV transmission risk, yes vs. no (ref)	0.59 (0.32–1.12)	0.098	0.57 (0.30–1.09)	0.080
Thinks IPV is justified, yes vs. no (ref)	0.24 (0.01–1.22)	0.170	0.26 (0.01–1.39)	0.205

Notes

a: OR—odds ratio; CI—confidence interval obtained with robust clustered standard errors.

## Discussion

In this peri-urban setting, lifetime IPV and excess fertility were 23% and 26%, respectively, and approximately 22% of couple responses were discordant on reported number of children they liked to have. There are no acceptable levels for IPV, and excess fertility is the result of couple’s own actual and desired fertility differences. Accordingly, these results suggest that the underlying factors associated with these outcomes may not have been adequately addressed. The communities we studied are relatively close to Blantyre City, a large urban center in Southern Malawi with tertiary services and are also close to an active local trading center and a long-standing family planning center that incorporates free services for both men and women. Our expectation was that the proximity of the site to these free services would have allowed for easier diffusion of information regarding reproductive health, and therefore the frequency of these adverse outcomes could be lower compared to previous reports [11].

The findings from this study are consistent with what has been observed in the Malawi national survey of 2015 [11] and other studies in Malawi [18]. We note that most of the available data in Malawi on IPV estimates and its determinants come from the MDHS; albeit this survey was completed approximately seven years ago, it remains an excellent source to provide aggregate and representative information at the national level. For example, Chikhungu et al conducted a cluster and multinomial logistic regression analyses using the MDHS from 2015 and reported that correlates of IPV significantly differ by levels of abuse and range from as low as 8.5% for high and complete abuse to as high as 27.2% for moderate physical and emotional abuse [12].

There are also similarities between our findings and data from earlier studies in Malawi. A separate national gender-based violence study among 3,546 females and 2,246 males reported in 2005 that 30% of women had physical abuse by a male partner; 28% had been economically abused (through withholding money); 25% had been emotionally abused; and 18% had been sexually abused. Among men in the same survey, 22% thought it was an acceptable behavior to limit movement of their partners outside the house; 20% thought it was acceptable to prevent their partner from communicating with outside individuals; less than 10% considered slapping or hitting their partner to be acceptable; and 14% reported that their female partner had attempted to prevent them from communicating with the outside. Physical violence was also experienced by men; in this study from 2005, 7% experienced slapping or hitting, and 6% experienced shoving by their partner [19].

Male partner alcohol consumption was a risk factor for both IPV and similarly for discordance in fertility preferences as reported by women. Similarly, Chikhungu et al, in a study in Malawi reported that alcohol consumption by the male partner was a risk factor for controlling behavior, physical, and emotional abuse [12]. A systematic review by Semahegn and Mengistie among Ethiopian women found that lifetime domestic physical violence (which ranged from 31 to 76.5% and lifetime domestic sexual violence from 19% to 59%) was associated with alcohol consumption and family history of violence was associated with physical or sexual violence against women [20]. Within the review, individual studies found odds ratios that ranged from 1.9–4.7, which is consistent with our adjusted odds ratio of 2.23. However, alcohol consumption and violence association is complex, as aside from its direct effects via reduced self-control, it could be a coping mechanism for abusive behavior, or reflective of other complex associations with socioeconomic and cultural variables [12,21]. Interestingly, we also noted a strong association between reported woman’s father’s practice of violence with woman’s mother being associated with her IPV experience; this may suggest an intergenerational cultural tolerance of this practice within this setting in Malawi [22–25].

Two factors were significantly associated with excess fertility in our study: increase in age and use of an effective family planning method. While a demographic explanation of older age association with excess fertility is plausible (as women age more children are expected), the association of excess fertility with use of an effective family planning method likely reflects a temporal bias (i.e., in this cross-sectional study, women with more children may be more likely to report current use of an effective method to prevent more pregnancies) [26]. We did not find potential alternative explanations such as differences in socio-demographic characteristics among users and non-users of an effective family planning method. We also analyzed factors associated with discordance in fertility preferences. None of the factors we examined showed a statistically significant association with fertility preference discordance among couples. However, among women we found a negative association between discordance and two factors: use of an effective family planning method and reported ability to refuse sex, potentially reflecting autonomy or ability to communicate with partner when making a reproductive health decision. We also found that men in monogamous relations were more likely to agree with their wives in fertility preference; this is in agreement with another study from northern Malawi where polygamous couples disagreed more than monogamous couples on future preferences regarding more children, and that use of contraceptive methods was less common in polygamous couples [27].

Our motivation in conducting this study emanates from a common observation we noticed over a period of 30 years of research in Malawi among women, mainly on the impact of HIV/AIDS [28,29]. Women of reproductive age consistently reported that they needed consent of their partners to participate in prevention and treatment studies–even when these activities involved health benefits for them, their children, and their spouses such as use of antiretrovirals to treat HIV and female microbicides to prevent acquisition of HIV. Similarly, women would not disclose their HIV status to their male partners because they were highly economically dependent on them for support or fear of retribution. Our findings shed light on the dynamics of family life in typical communities around Blantyre, Malawi, and the need for additional work to educate members of the community to eliminate IPV and reduce unwanted fertility, though current results do not identify shared risk factors for these important outcomes. Our study was cross-sectional and not different from the MDHS or other surveys, and therefore share a common limitation of being a one-time inquiry where directionality cannot be ascertained when examining associations or assessing trends over time. Nonetheless, this localized survey targeted specific communities straddling rural and urban life and confirmed that gender-based adverse outcomes remain common, irrespective of the geographic setting. Therefore, bold initiatives involving both governmental and non-governmental programs should prioritize gender equity activities through communication, education at all levels (including traditional leaders), and enforcement of existing and new measures.

## Conclusions

In examining key gendered health outcomes of IPV, excess fertility, and spousal discordance on fertility preferences, current partner alcohol consumption emerged as a shared correlate of lifetime IPV, and with spousal discordance on fertility preferences. Given the temporal sequencing limitations of our cross-sectional design, it remains unclear whether alcohol is a true modifiable risk factor or potentially a consequence of these experiences. Our estimates of the frequency of IPV and excess fertility are mostly comparable to the MDHS data from Southern Malawi; relative differences may reflect changing population structure and trends over time (the MDHS was conducted more than five years ago). More importantly, our data as well as findings from other studies show persistent socio-cultural problems that need further attention. We suggest future interventions to promote gender equity should not exclusively target women, but also consider empowerment of the couple to decrease violence towards women and promote shared decisions regarding fertility and measures such as family planning that can benefit the couple and well-being of the family and community.

## Supporting information

S1 File (DOCX)

S2 File (XLSX)

S3 File (XLSX)

S4 File (XLSX)

S5 File (XLSX)
